# Efficacy and safety evaluation of frontline immunotherapy combinations in advanced esophageal squamous cell carcinoma: a network meta-analysis highlighting the value of PD-L1 expression positivity scores

**DOI:** 10.3389/fimmu.2024.1414753

**Published:** 2024-07-10

**Authors:** Wei Chen, Keming Cao, Lili Zhang, Xingyu Zhao, Bixiao Chen, Wei Li, Rongguo Shang, Lichaoyue Sun, Ze Jiang, Jingxin Wang, Wenxin Xue

**Affiliations:** ^1^ Department of Pharmacy, Emergency General Hospital, Beijing, China; ^2^ School of Pharmacy, North China University of Science and Technology, Heibei, China; ^3^ School of Pharmaceutical Sciences, Capital Medical University School, Beijing, China; ^4^ Beijing Shijitan Hospital, Capital Medical University, Beijing, China; ^5^ Pharmacy Department, Aerospace Center Hospital, Beijing, China; ^6^ Pharmaceutical Department, TongRen Hospital, Capital Medical University, Beijing, China

**Keywords:** esophageal squamous cell carcinoma, immune checkpoint inhibitors, efficacy, safety, network meta-analysis

## Abstract

**Introduction:**

The systematic review and network meta-analysis (NMA) consolidate all relevant randomized controlled trials (RCTs) related to initial immunotherapy treatments for advanced esophageal squamous cell carcinoma (ESCC). Our goal is to thoroughly assess the effectiveness and safety of various immunotherapy methods, focusing on overall survival (OS) and progression-free survival (PFS) among patients with advanced ESCC positive for PD-L1.

**Methods:**

We conducted a systematic search of the PubMed, Embase, Cochrane Library, and Web of Science databases, covering all records from their inception until January 22, 2024. The inclusion criteria targeted patients with advanced ESCC undergoing first-line immunotherapy or chemotherapy, limiting the study selection to randomized controlled trials (RCTs) exclusively. The study upholds the values of openness, originality, and dependability, as evidenced by its enrollment in the Prospective Register of Systematic Reviews (CRD42024504992).

**Results:**

Our analysis encompasses 7 RCTs, totaling 4688 patients, and evaluates 8 distinct immunotherapy combinations. In advanced ESCC patients irrespective of PD-L1 expression, both sintilimab-chemotherapy and toripalimab-chemotherapy regimens demonstrated comparable OS benefits (HR=0.92, 95% CI: 0.64-1.33). The most pronounced PFS advantages were seen with sintilimab-chemotherapy and camrelizumab-chemotherapy as compared to standard chemotherapy (HR=0.56, 95% CI: 0.46-0.58). Notably, camrelizumab-chemotherapy (HR=0.83, 95% CI: 0.59-1.16) and nivolumab-ipilimumab (HR=0.84, 95% CI: 0.60-1.17) demonstrated significant safety profiles over chemotherapy alone. Subgroup analysis based on PD-L1 expression revealed nivolumab-chemotherapy to yield the highest OS benefit (HR=0.54, 95% CI: 0.37-0.79) in ESCC patients with PD-L1 expression ≥1%. Furthermore, camrelizumab-chemotherapy (HR=0.51, 95% CI: 0.39-0.67) exhibited superior PFS benefits. Among patients with PD-L1 expression ≥10%, camrelizumab-chemotherapy (HR=0.52, 95% CI: 0.35-0.78) emerged as the most efficacious in improving OS, while serplulimab-chemotherapy (HR=0.48, 95% CI: 0.34-0.68) was associated with the longest PFS benefit.

**Conclusion:**

The integration of immune checkpoint inhibitors (ICIs) with chemotherapy appears to significantly enhance survival outcomes in patients with advanced ESCC compared to chemotherapy alone. Sintilimab-chemotherapy is potentially the optimal regimen for patients without PD-L1 expression. In contrast, nivolumab-chemotherapy and camrelizumab-chemotherapy are likely to offer the best OS and PFS benefits, respectively, in patients with PD-L1 expression ≥1%. Among those with PD-L1 expression ≥10%, camrelizumab-chemotherapy is projected to provide the greatest OS advantage, whereas serplulimab-chemotherapy is anticipated to offer the most prolonged PFS benefit. Since most of the patients in this study originated from Asia, the above findings are more applicable to the Asian population.

**Systematic review registration:**

https://www.crd.york.ac.uk/prospero/, identifier CRD42024504992.

## 1 Introduction

Worldwide, esophageal cancer (EC) ranks as the seventh most frequently identified cancer and the sixth top cancer mortality cause, predominantly occurring in Asia and Africa (1). ESCC, the dominant subtype in Asian populations, comprises 90% of EC cases (2) and represents over half of the global ESCC burden, with a significant prevalence in China (3). The prognosis for ESCC is notably grim, largely attributed to its advanced or metastatic state at diagnosis, which is the case for 60%-70% of patients. This results in a dismal five-year OS rate of less than 15% (4, 5). Standardized chemotherapy regimens for advanced or metastatic ESCC have been explored for decades, with fluoropyrimidine and platinum-based regimens constituting the standard first-line systemic therapy (6–8). Paclitaxel, docetaxel, or irinotecan monotherapy is often employed as second-line therapy for advanced or metastatic ESCC (9, 10). However, the efficacy of both first-line and second-line chemotherapy remains unsatisfactory, with OS typically less than 10 months (11, 12). The limited efficacy of chemotherapy, coupled with its significant side effects, severely impacts patients’ quality of life. In light of the frequent occurrence of advanced ESCC and unfavorable treatment results, scientists are actively working to create innovative treatment approaches to extend patient lifespans.

The advent of ICIs has offered new potential in cancer treatment. These agents work by binding to proteins on T cells, reinvigorating T cell activity, and bolstering the body’s immune response against tumors (13, 14). Key immune checkpoints in ESCC, such as Programmed cell death protein 1 (PD-1), programmed death-ligand 1 (PD-L1), and cytotoxic T-lymphocyte antigen-4 (CTLA-4), are targeted by monoclonal antibodies, producing therapeutic effects (15). Extensive randomized controlled trials like Keynote-590 and Checkmate 648 have shown that combining PD-1 inhibitors with chemotherapy is more effective than solely chemotherapy as an initial treatment for advanced ESCC (16, 17). Consequently, the National Comprehensive Cancer Network (NCCN) now recommends a PD-1 inhibitor in combination with chemotherapy as a primary treatment strategy (18).

With the growing number of RCTs exploring ICIs, predominantly comparing them in combination with chemotherapy against standard chemotherapy, there is a pressing need to determine the most effective immune combination therapy strategies. This is vital for guiding the design of future head-to-head clinical trials.

Our study seeks to assess the efficacy and safety of all currently available first-line immune combination therapy regimens for patients with advanced ESCC. We employ a Bayesian framework for indirect comparison of each regimen’s efficacy and safety, aiming to ascertain the most optimal treatment options across different levels of PD-L1 expression through a systematic review and meta-analysis.

## 2 Materials and methods

This NMA rigidly follows to the Preferred Reporting Items for Systematic Reviews and Meta-Analysis (PRISMA) extension statement for NMAs, as detailed in **Supplementary Table 1** (19). The absence of direct comparisons in RCTs involving various immunotherapy combinations necessitates the use of Bayesian methods. These methods provide a probabilistic framework conducive to indirect comparison, thereby enabling predictions regarding the efficacy and safety of different treatments (20). Highlighting its dedication to openness, dependability, and creativity, the research protocol is listed in the Prospective Register of Systematic Reviews (CRD42024504992).

### 2.1 Data sources and search strategy

The research led to an extensive and detailed exploration spanning major databases such as PubMed, EMBASE, the Cochrane Library, and Web of Science. The search engine we employed included various key terms: ‘esophageal squamous cell carcinoma,’ ‘oesophageal squamous cell carcinoma,’ ‘randomized clinical trial,’ ‘immune checkpoint inhibitors,’ ‘PD-L1 inhibitor,’ ‘PD-1 inhibitor,’ ‘CTLA-4 Inhibitor,’ along with the identification of specific ICIs like ‘sintilimab,’ ‘pembrolizumab,’ ‘toripalimab,’ ‘camrelizumab,’ ‘nivolumab,’ ‘ipilimumab’, ‘serplulimab,’ and ‘tislelizumab.’ Further specifics on the chosen keywords are outlined in **Supplementary Table 2**. The scope of our literature review extended from the inception of each database until January 22, 2024, encompassing both unstructured and regulated vocabulary expressions to guarantee a comprehensive and targeted collection of pertinent research. The methodology was crafted to capture the latest and most detailed information obtainable, in harmony with the stringent criteria set by high-caliber systematic reviews and meta-analyses in the oncology realm.

### 2.2 Selection criteria

Inclusion Criteria:

(1) RCTs enrolling patients with advanced ESCC, verified through histological or cytological analysis.
(2) RCTs assessing the efficacy of ICIs when combined with chemotherapy, serving as a primary treatment approach.
(3) RCTs comparing the therapeutic impact of ICIs in conjunction with chemotherapy against alternative treatment strategies for advanced ESCC.
(4) RCTs reporting at least one of the following outcome measures:
  -Overall Survival is measured by the time span from enrolling in the study to death due to any cause.
  -Progression-Free Survival is measured by the time span from joining the study to either the advancement of the illness or death due to any reason.
  - Grade 3 or higher AEs.

Exclusion Criteria:

(1) RCTs involving different stages of the disease in the same group of patients.
(2) RCTs with unclear outcome measures.
(3) Reviews or case reports.

The initial screening of RCTs was performed by examining titles and abstracts. Subsequently, a rigorous dual-review procedure was implemented, involving two independent researchers to confirm the inclusion of the most recent and relevant data.

### 2.3 Data extraction and quality assessment

Three separate evaluators carefully gathered data from the RCTs, complying with the standards set by the Preferred Reporting Items for Systematic Reviews and Meta-Analyses (PRISMA) framework. Discrepancies in interpreting the data were resolved by engaging in a consultative dialogue with a fourth author. The data extraction process encompassed a comprehensive range of trial characteristics, including the trial name, design specifications, randomization ratio, source and year of publication, trial phase, tumor stage, ClinicalTrials.gov identifier, cohort size, demographic breakdown (age and gender distribution), histological classification, patient ethnicity, PD-L1 expression levels, Eastern Cooperative Oncology Group (ECOG) performance status, current disease state, and detailed descriptions of the treatment protocols for both the experimental and control groups. Additionally, critical outcome metrics were collated, notably the Hazard Ratios (HRs) and the associated 95% Confidence Intervals (CIs) for OS and PFS, along with incidences of Grade 3 or more Adverse Events.

The assessment of methodological soundness and possible biases in the incorporated RCTs utilized the Cochrane Risk of Bias Tool 2.0. The tool offered conducts a comprehensive analysis in five separate areas: the likelihood of bias during the randomization phase, bias arising from straying from planned interventions, bias from not thoroughly reported outcomes, bias impacting the unbiased assessment of outcomes, and bias in result disclosure. This thorough analysis classifies the RCTs involved into three unique risk profiles: low risk, high risk, and presenting ‘some concerns’.

### 2.4 Statistical analysis

During the study, OS and PFS were the primary outcomes, while Grade 3 or more Adverse Events were identified as the secondary ones. The research utilized Hazard Ratios along with corresponding 95% Confidence Intervals to assess their impacts on Overall Survival and Progression-Free Survival. In response, we employed Odds Ratios (ORs) and 95% CIs for assessing the occurrence of Grade 3 or more severe AEs.

Using a Bayesian framework, a NMA was executed with the ‘rjags’ and ‘gemtc’ tools in R to assess the effectiveness and safety of combined frontline immunotherapy in managing Advanced ESCC. The study included three separate Markov chains within a fixed-effects framework, conducting 20,000 initial burn-in phases and subsequently 50,000 sampling cycles each. The aggregated results for HRs and ORs from these Markov chains facilitated the ranking of various treatment regimens in terms of efficacy and safety. These rankings were subsequently illustrated through comprehensive graphical representations.

For scenarios lacking direct comparison trials, indirect comparisons were executed through NMA. The reliability of these indirect comparisons was corroborated by conducting pairwise meta-analyses employing frequentist approaches for directly comparable studies. The findings from these analyses were juxtaposed against summary results obtained from the Bayesian NMA (refer to **Supplementary Table 3** for detailed outcomes).

Furthermore, pairwise meta-analyses were conducted utilizing Revman 5.4 software, adopting frequentist methodologies, to ascertain the overall efficacy and safety of first-line immunotherapy combinations versus monotherapy. Heterogeneity evaluation utilized the Q-test and *I^2^* statistic, with *I^2^* ≤ 50% or *P* ≥ 0.1 indicating low heterogeneity and *I^2^* > 50% or *P* < 0.1 signifying high heterogeneity. Studies characterized by significant heterogeneity were analyzed using random-effects models, whereas those with minimal heterogeneity were evaluated using fixed-effects models. Sensitivity analyses were performed for highly heterogeneous studies by sequentially excluding studies exerting a substantial influence on heterogeneity, to assess the consistency of summary efficacy and safety outcomes. The assessment of publication bias was conducted using funnel plot analysis, setting the statistical significance threshold at α = 0.05.

## 3 Results

### 3.1 Systematic review and characteristics

**Figure 1:**
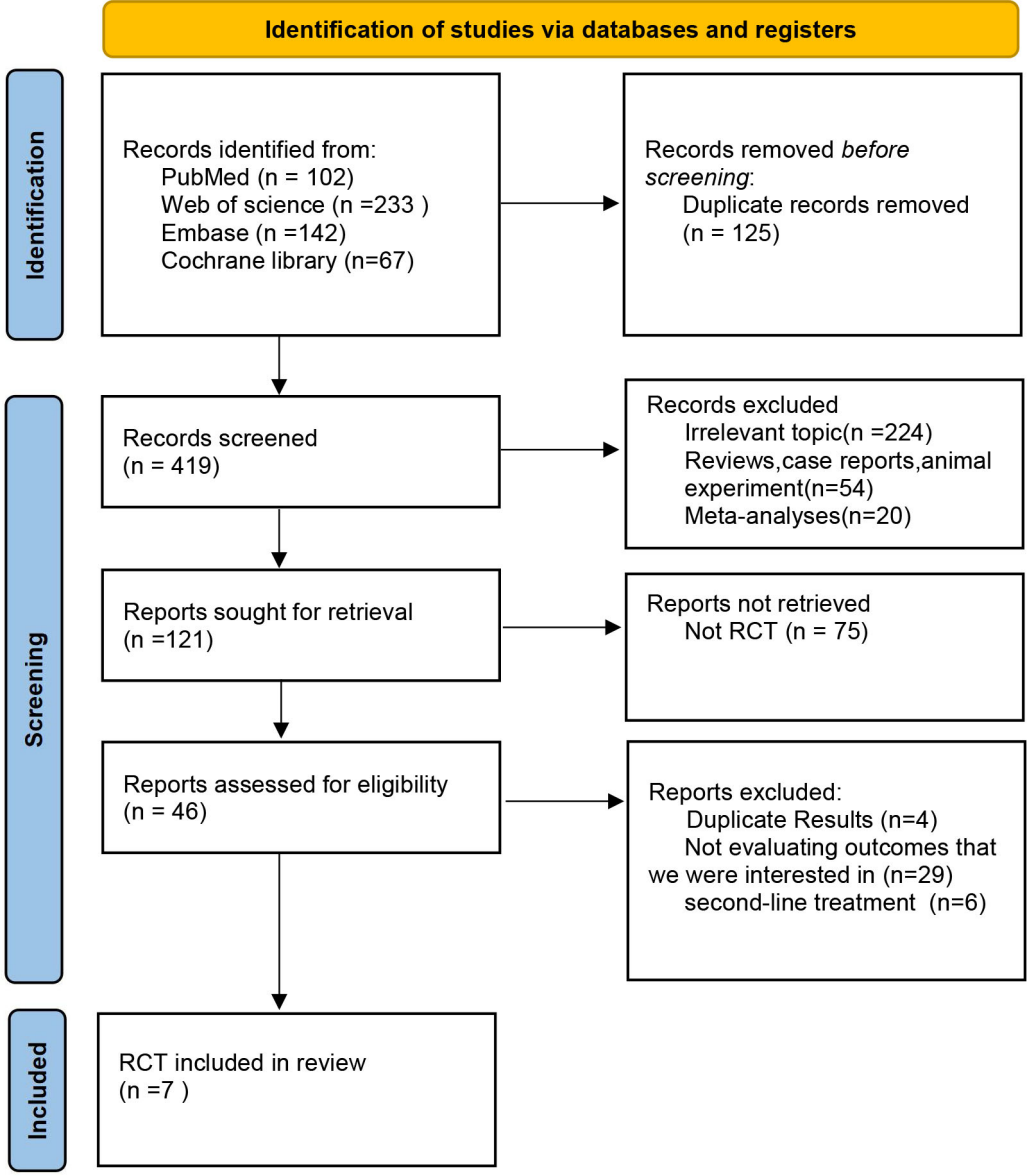
Flowchart of Literature Search and Screening Process. This study followed the PRISMA guidelines for systematic reviews and meta-analyses. Life Science Identifiers.

In the initial phase of our literature review, a comprehensive search across multiple databases yielded a total of 544 records. Subsequent abstract screening for the removal of duplicates and articles not pertinent to our research focus resulted in 419 studies being selected for detailed full-text evaluation. After a thorough review, only 7 studies satisfied our stringent inclusion criteria (refer to **Figure 1**). The risk assessment diagram for bias can be found in **Supplementary Figure 1**. These studies encompassed a collective patient cohort of 4,688 individuals, undergoing treatment across nine distinct regimens: Sintilimab plus chemotherapy (sinti-chemo), Pembrolizumab plus chemotherapy (pem-chemo), Toripalimab plus chemotherapy (toripa-chemo), Camrelizumab plus chemotherapy (camre-chemo), Nivolumab plus chemotherapy (nivo-chemo), Nivolumab plus Ipilimumab (nivo-ipi), Serplulimab plus chemotherapy (serplu-chemo), Tislelizumab plus chemotherapy (tisle-chemo), and chemotherapy alone (chemo). Comprehensive details regarding the methodology, patient demographics, and outcomes of these studies are systematically cataloged in **Tables 1**, **2**, and **Supplementary Table 4** (16, 17, 21–25). **Supplementary Figure 1** details the risk assessment of bias in the studies included.

**Table 1:** Baseline characteristics of studies included in the network meta-analysis.

Study	source	Registered ID	Sample Size	Stage				
(Phase, Design)	(y)	(Randomization)	(Median Age/y)	(Male/ Female)	Histology	Ethnicity (%)	Intervention Arm(s)	Control Arm
ORIENT-15	BMJ	NCT03748134	327/332	III-IV	Squamous	Asian (97.3) White (1.8)	Sintilimab 3mg/kg (<60kg) or 200mg (>60kg) Q3W	placebo 3mg/kg (<60kg) or 200mg (>60kg) Q3w
(double-blind,III)	2022	(1:1)	(63/63)	567/92		Not reported (0.9)	**+**Cisplatin (75 mg/m2 Q3W) **+**paclitaxel (cycle 1:87.5mg/m^2^ on day1/8;other cycle 175 mg/m^2^ Q3W) **or** 5-fluorouracil (800 mg/m^2^ Q3W)	**+**Cisplatin (75 mg/m2 Q3W) **+**paclitaxel (cycle 1:87.5mg/m^2^ on day1/8;other cycle 175 mg/m^2^ Q3W) **or** 5-fluorouracil (800 mg/m^2^ Q3W)
KEYNOTE-590	Lancet	NCT03189719	373/376	IV	Squamous	Asian (53.4) White (37.1)	pembrolizumab 200 mg	saline placebo
(double-blind,III)	2021	(1:1)	(64/62)	625/124		Missing (3.9) Native American (2.8) African American (0.9) Other (1.9)	**+**5-fluorouracil 800 mg/m² on days 1–5 **+**cisplatin 80 mg/m² on day 1 (for a maximum of six cycles)	**+**5-fluorouracil 800 mg/m² on days 1–5 **+**cisplatin 80 mg/m² on day 1 (for a maximum of six cycles)
JUPITER-06	Cancel Cell	NCT03829969	257/257	IV	Squamous	China (100.0)	toripalimab (240 mg) Q3W	placebo Q3W
(double-blind,III)	2022	(1:1)	(63/62)	437/77			**+**paclitaxel (175 mg/m^2^) Q3W and cisplatin (75 mg/m2) Q3W	**+**paclitaxel (175 mg/m^2^) Q3W and cisplatin (75 mg/m^2^) Q3W
ESCORT-1st	JAMA	NCT03691090	298/298	IV	Squamous	China (100.0)	Camrelizumab (200 mg) Q3W	placebo Q3W
(double-blind,III)	2021	(1:1)	(62/62)	523/73			**+**Paclitaxel(175 mg/m^2^) Q3W **+**cisplatin (75 mg/m^2^) Q3W	**+**Paclitaxel (175 mg/m^2^) Q3W **+**cisplatin (75 mg/m^2^) Q3W
Checkmate 648	NEJM	NCT03143153	321/325 /324	IV	Squamous	Asian (70.6) White (25.6)	Arm1:nivolumab (240mg) Q2W **+**fluorouracil (800mg/m^2^) **+**cisplatin (80mg/m^2^)	fluorouracil (800mg/m^2^) day1-5 **+**cisplatin (80mg/m^2^) day1
(open-label,III)	2022	(1:1:1)	(64/63/64)	797/173		Black (1.1) Other (2.7)	Arm2: nivolumab(3mg/m2) Q2W **+**ipilimumab(1mg/kg) Q6W	
ASTRUM-007	NAT MED	NCT03958890	368/183	III-IV	Squamous	China (100.0)	serplulimab (3mg/kg) on day1,Q2W	placebo on day1,Q2W
(double-blind,III)	2023	(2:1)	(64/64)	470/81			**+**cisplatin (50 mg/m^2^) on day1,Q2W **+**5-fluorouracil (1200 mg/m^2^) (on days1and2), Q2W	**+**cisplatin (50 mg/m^2^) on day1,Q2W **+**5-fluorouracil (1,200 mg/m^2^) (on days1、2), Q2W
RATIONALE 306	Lancet Oncol	NCT03783442	326/323	IV	Squamous	Asian (74.9) White (23.9)	Tislelizumab (200 mg) Q3W	placebo Q3W
(double-blind,III)	2023	(1:1)	(64/65)	563/86		American Indian or Alaska Native (0.1) Not reported or unknown (1.1)	**+**cisplatin 60–80 mg/m² (or oxaliplatin 130 mg/m²) Q3W **+**fluorouracil (750–800 mg/m²) on days 1–5] **or** capecitabine 1000 mg/m² po.bid. on day1-14) **or** paclitaxel (175 mg/m²) Q3W	**+**cisplatin 60–80 mg/m² (or oxaliplatin 130 mg/m²) Q3W **+**fluorouracil (750–800 mg/m²) on days 1–5] **or** capecitabine 1000 mg/m² po.bid. on day1-14) **or** paclitaxel (175 mg/m²) Q3W

**Table 2:** Characteristics of Included Randomized Controlled Trials.

Study	PD-L1 Detection	PD-L1≥1% Patients (%)			PD-L1 ≥10% Patients (%)		Reported Outcomes
		Intervention(s),n (%)		Control, n (%)	Intervention(s),n(%)	Control, n (%)	
**ORIENT-15**	CPS、TPS	295 (90)		309(93)	188 (57)	193 (58)	OS, PFS, grade≥3 AEs
**KEYNOTE-590**	CPS	/		/	186 50)	197 (52)	OS, PFS, grade≥3 AEs
**JUPITER-06**	CPS	201 (78.2)		200(77.8)	115 (44.7)	97 (37.7)	OS, PFS, grade≥3 AEs
**ESCORT-1st**	TPS	166 (55.7)		163(54.7)	104 (34.9)	98 (32.9)	OS, PFS, grade≥3 AEs
**Checkmate 648**	TPS	158 (49)	158 (49)	157(48)	/	/	OS, PFS, grade≥3 AEs
**ASTRUM-007**	CPS	/		/	162 (44)	79 (43)	OS, PFS, grade≥3 AEs
**RATIONALE 306**	CPS	/		/	116 (36)	107 (33)	OS, PFS, grade≥3 AEs

### 3.2 Pairwise meta-analysis

#### 3.2.1 Comparisons of OS, PFS

**Figure 2:**
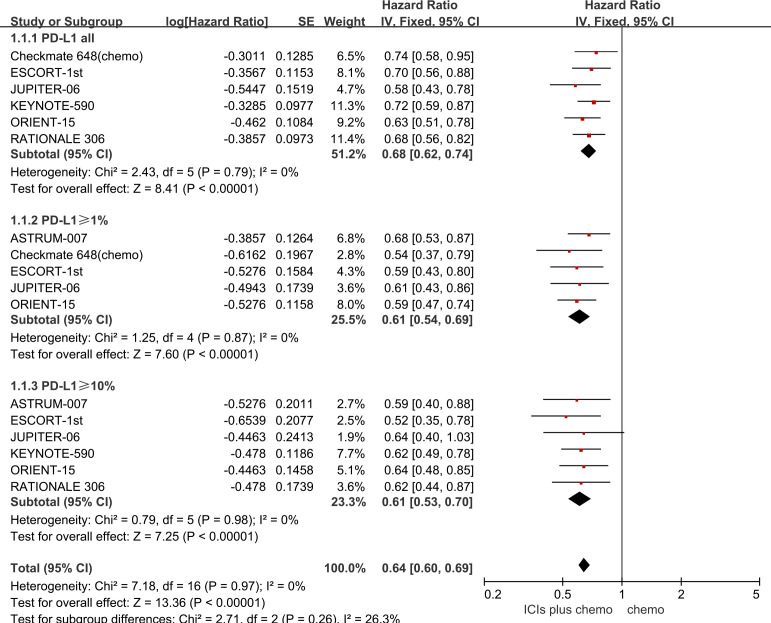
Forest plot comparing the use of ICIs combined with chemotherapy versus chemotherapy alone for OS in ESCC patients, stratified by PD-L1 expression levels: no PD-L1 expression, PD-L1 expression ≥1%, and PD-L1 expression ≥10%.

All seven studies included in this meta-analysis reported OS outcomes, and no statistically significant heterogeneity was observed among them (*P* > 0.1, *I^2 =^* 0). A fixed-effects model was employed for the meta-analysis. The results demonstrate that the combined use of ICIs with chemotherapy significantly prolongs OS compared to chemotherapy alone, irrespective of PD-L1 expression levels in patients with ESCC. For ESCC patients without PD-L1 expression (HR = 0.68, 95% CI: 0.62-0.74), those with PD-L1 expression ≥1% (HR = 0.61, 95% CI: 0.54-0.69), and those with PD-L1 expression ≥10% (HR = 0.64, 95% CI: 0.60-0.69), the combination therapy yielded significant OS benefits. Refer to **Figure 2** for details.

**Figure 3:**
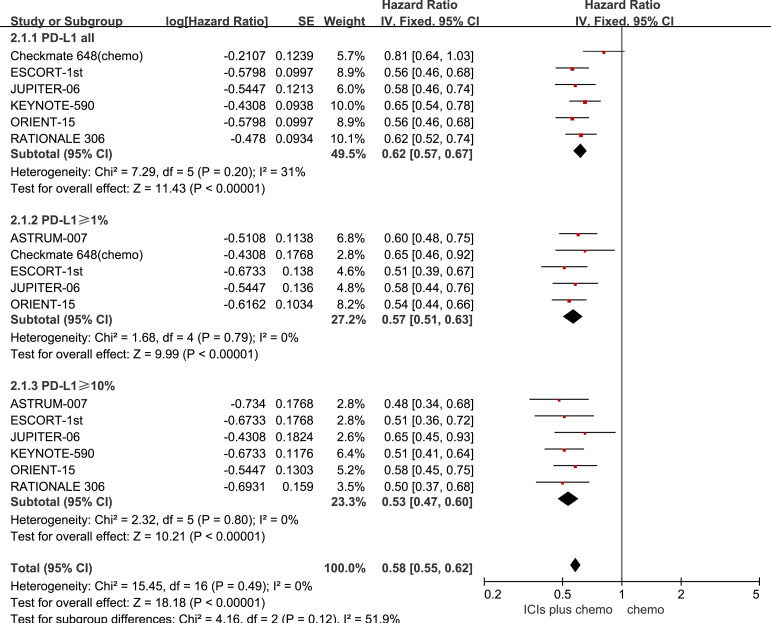
Forest plot comparing the use of ICIs combined with chemotherapy versus chemotherapy alone for PFS in ESCC Patients, stratified by PD-L1 expression levels: no PD-L1 expression, PD-L1 expression ≥1%, and PD-L1 expression.

All seven studies reported PFS, with no statistically significant heterogeneity observed among them (*P* > 0.1, *I^2^* < 50%). A fixed-effects model was used for the meta-analysis. The results indicate that the combined use of ICIs with chemotherapy significantly prolongs PFS compared to chemotherapy alone, regardless of PD-L1 expression status in patients with ESCC. For ESCC patients without PD-L1 expression (HR = 0.62, 95% CI: 0.57-0.67), those with PD-L1 expression ≥1% (HR = 0.57, 95% CI: 0.51-0.63), and those with PD-L1 expression ≥10% (HR = 0.53, 95% CI: 0.47-0.60), the combination therapy yielded significant PFS benefits. See **Figure 3** for details.

#### 3.2.2 Safety and toxicity

**Figure 4:**
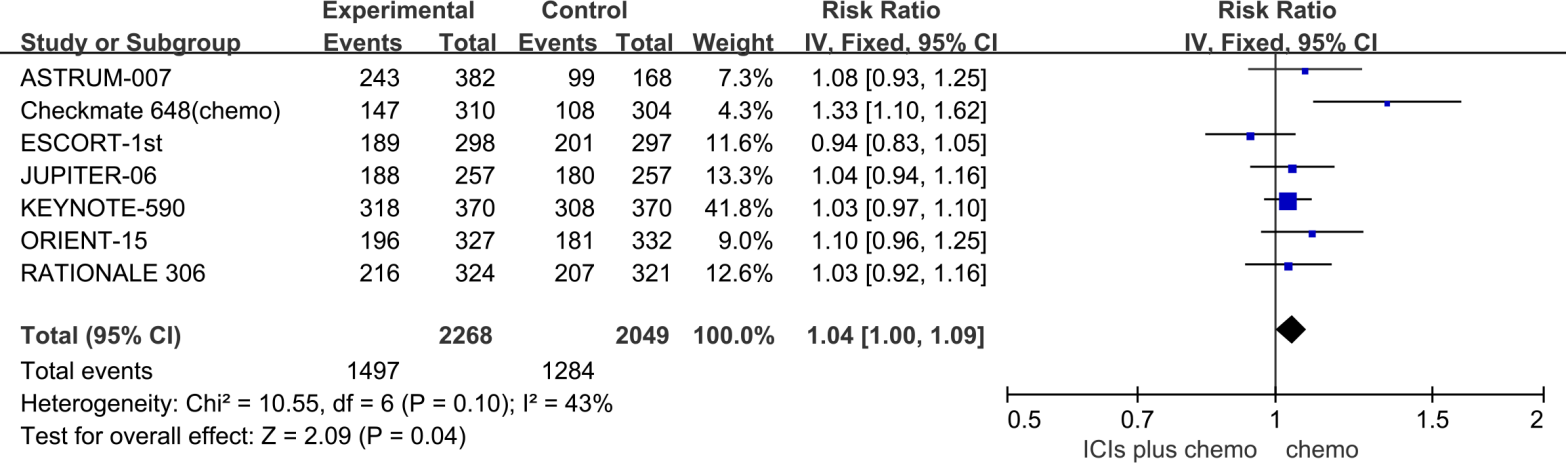
Forest plot comparing the use of ICIs combined with chemotherapy versus chemotherapy alone for the incidence of grade 3 or higher AEs in Patients with Advanced ESCC.

The incidence of AEs with a severity of grade 3 or higher was evaluated to assess the safety and toxicity of ICIs combined with chemotherapy. All seven studies reported the incidence of grade 3 or higher AEs, with minimal statistical heterogeneity among them (*P*=0.1, *I^2^ =* 43%), and a fixed-effects model was employed for the meta-analysis. The results indicate that for patients with advanced ESCC, the use of ICIs combined with chemotherapy leads to a higher incidence of grade 3 or higher AEs compared to chemotherapy alone (RR=1.04, 95%CI: 1.00-1.09). Refer to **Figure 4** for details.

### 3.3 Network meta-analyses

#### 3.3.1 Comparisons of OS, PFS

**Figure 5:**
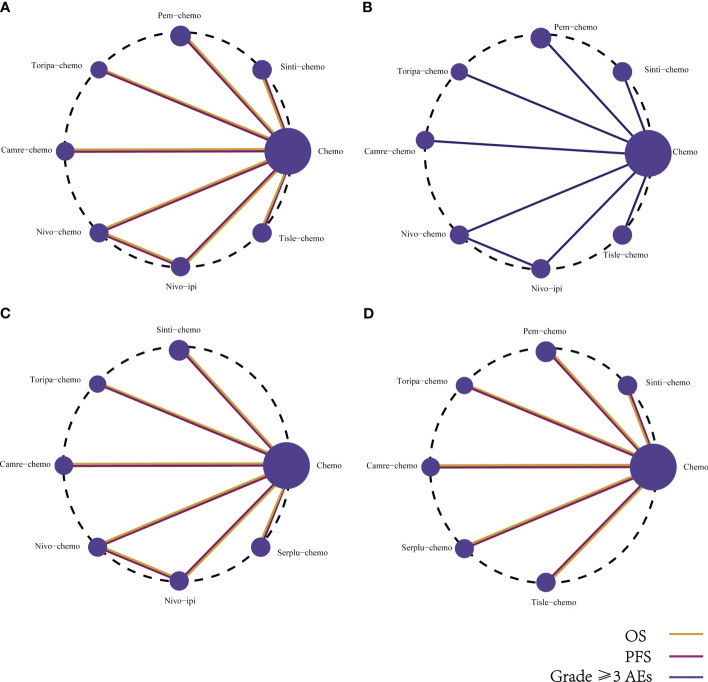
Network diagram comparing the efficacy and safety of various immunotherapy regimens in late-stage ESCC patients with different PD-L1 expressions. The network diagram illustrates comparisons conducted utilizing the Bayesian framework regarding **(A)** OS, PFS, and **(B)** AEs of grade ≥3 in patients without PD-L1 selection. Additionally, comparisons of OS and PFS are presented for late-stage ESCC patients with **(C)** PD-L1 expression ≥1%, and **(D)** PD-L1 expression ≥10%.

The primary endpoints of this study encompassed OS and PFS. Our NMA incorporated eight immunotherapy combination regimens for advanced ESCC patients without preselection for PD-L1 expression (**Figure 5A**).

**Figure 6:**
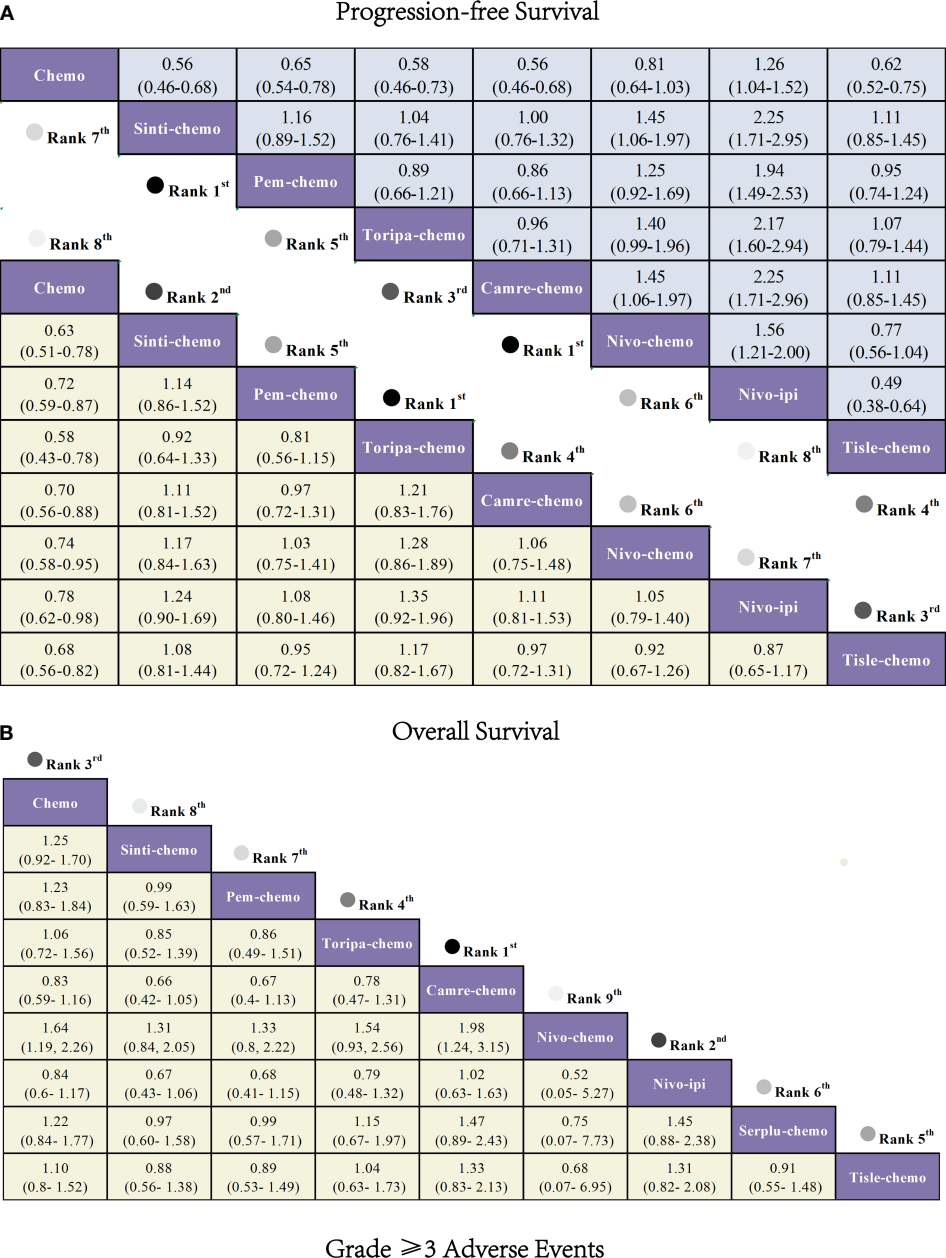
Forest plot comparing the efficacy and safety of various immunotherapy regimens in late-stage ESCC Patients without PD-L1 selection based on Bayesian network meta-analysis. **(A)** Risk ratios and 95% CIs for OS (depicted by the yellow lower triangle area) and PFS (depicted by the blue upper triangle area). HR < 1.00 indicates a superior survival benefit. **(B)** ORs and 95% CIs for grade 3 AEs. OR < 1.00 indicates better safety.

Regarding OS (**Figure 6A**), patients receiving combination immunotherapy exhibited a higher likelihood of experiencing greater OS benefits compared to those undergoing standard chemotherapy. Among these, toripa-chemo demonstrated the most optimal OS benefit (HR = 0.58, 95% CI: 0.43-0.78) when compared to chemotherapy. Furthermore, the study observed comparable OS benefits between sinti-chemo and toripa-chemo (HR = 0.92, 95% CI: 0.64-1.33). Additionally, there were no significant OS benefits observed when comparing sinti-chemo to tisle-chemo (HR = 1.08, 95% CI: 0.81-1.44), camre-chemo (HR = 1.11, 95% CI: 0.81-1.52), pem-chemo (HR = 1.14, 95% CI: 0.86-1.52), nivo-chemo (HR = 1.17, 95% CI: 0.84-1.63), and nivo-ipi (HR = 1.24, 95% CI: 0.90-1.69).

In terms of PFS (**Figure 6A**), combination immunotherapy demonstrated superior PFS compared to standard chemotherapy. The only exception was nivo-ipi, which exhibited the poorest PFS among all treatment regimens. Within combination immunotherapy, sinti-chemo and camre-chemo similarly provided the best PFS benefits compared to chemotherapy (HR = 0.56, 95% CI: 0.46-0.58), followed by toripa-chemo (HR = 0.58, 95% CI: 0.46-0.73) and tisle-chemo (HR = 0.62, 95% CI: 0.52-0.75). Furthermore, significant PFS benefits were observed when comparing pem-chemo (HR = 0.65, 95% CI: 0.54-0.78) and nivo-chemo (HR = 0.81, 95% CI: 0.64-1.03) to chemotherapy.

#### 3.3.2 Safety and toxicity

Determining safety and toxicity is based on grade 3 and above AEs. The NMA encompassed eight combination regimens of ICIs associated with grade 3 and above AEs (**Figure 5B**).

**Figure 7:**
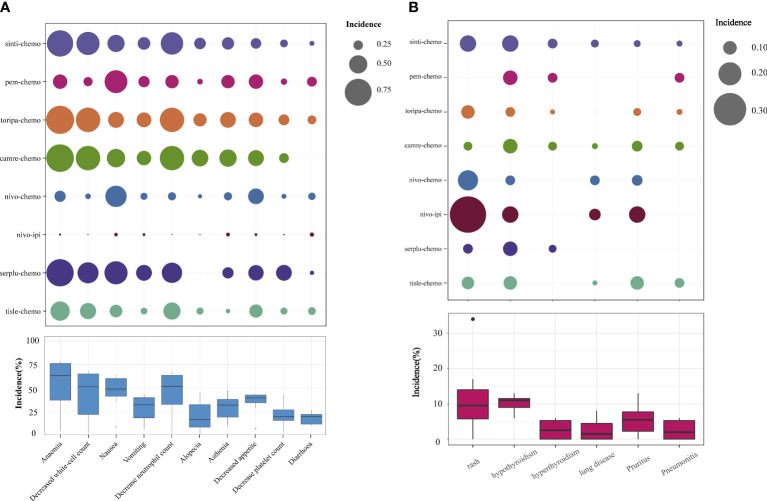
Safety Profile Characteristics of Various Immunotherapy Regimens. **(A)** Incidence of Treatment-Related Grade ≥3 AEs. **(B)** Incidence of Immunotherapy-Induced Grade ≥3 AEs.

Regarding grade≥3 adverse events (**Figure 6B**), only camre-chemo (HR = 0.83, 95% CI: 0.59-1.16) and nivo-ipi (HR = 0.84, 95% CI: 0.60-1.17) exhibited significant safety benefits compared to chemotherapy. Furthermore, toripa-chemo (HR = 1.06, 95% CI: 0.72-1.56), tisle-chemo (HR = 1.10, 95% CI: 0.80-1.52), serplu-chemo (HR = 1.22, 95% CI: 0.84-1.77), pem-chemo (HR = 1.23, 95% CI: 0.83-1.84), sinti-chemo (HR = 1.25, 95% CI: 0.92-1.70), and nivo-chemo (HR = 1.64, 95% CI: 1.19-2.26) showed no safety benefits compared to chemotherapy. No new safety adverse events were observed. Commonly reported treatment-related AEs in the ICIs combination therapy group included anemia, decreased white-cell count, nausea, vomiting, decreased neutrophil count, alopecia, asthenia, decreased appetite, decrease platelet count, and diarrhea. Immune-mediated AEs frequently reported in the ICIs combination therapy group included rash, hypothyroidism, hyperthyroidism, Immune-mediated lung disease, Pruritus, and Pneumonitis (**Supplementary Table 5**). The probability of different AEs varied across different ICIs combination therapy regimens. Camre-chemo was most likely to cause decreased white-cell count, decreased neutrophil count, alopecia, asthenia, and decreased appetite. Conversely, nivo-ipi exhibited the lowest associated risks for anemia, decreased white-cell count, nausea, vomiting, decreased neutrophil count, alopecia, asthenia, decreased appetite, decrease platelet count, and diarrhea (**Figure 7A**). The incidence rates of treatment-related adverse events (such as anemia, decreased white-cell count, decrease neutrophil count) varied significantly across different treatment regimens, while the spectrum of occurrence rates for immune-mediated adverse events (such as hypothyroidism, pneumonitis) was narrower (**Figure 7B**; **Supplementary Table 5**).

### 3.4 Rankings

**Figure 8:**
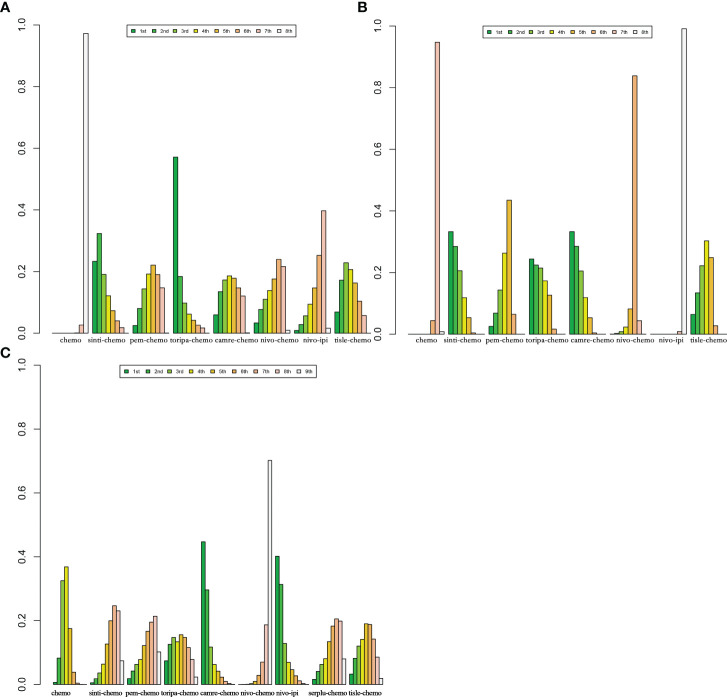
Bayesian ranking features of efficacy and safety of various immunotherapy regimens in advanced ESCC Patients without PD-L1 Selection. **(A)** Ranking of OS. **(B)** Ranking of PFS. **(C)** Ranking of Grade ≥3 AEs.

According to the Bayesian rank probability analysis (**Figure 8**; **Supplementary Table 6**), for ESCC patients without selected PD-L1 expression, toripa-chemo is most likely to rank first in terms of OS with a cumulative probability of 57.12%. Camre-chemo ranks first in PFS with a probability of 33.29%, and it also ranks first in grade ≥3 AEs with a probability of 44.67%. Nivo-chemo demonstrates the highest probability (70.16%) of causing grade ≥3 AEs. Camre-chemo achieves an effective balance between efficacy and safety, as it ranks fifth (17.85%) in OS, first (33.29%) in PFS, and first (44.67%) in grade ≥3 AEs.

### 3.5 Subgroup analysis

#### 3.5.1 On the basis of PD-L1 expression level

Using OS and PFS as outcome measures, the study investigated the outcomes of advanced ESCC patients with different levels of PD-L1 expression, categorized as ≥1% and ≥10%. The optimal ICIs combination therapies differed between these two subgroups (**Supplementary Table 8**).

**Figure 9:**
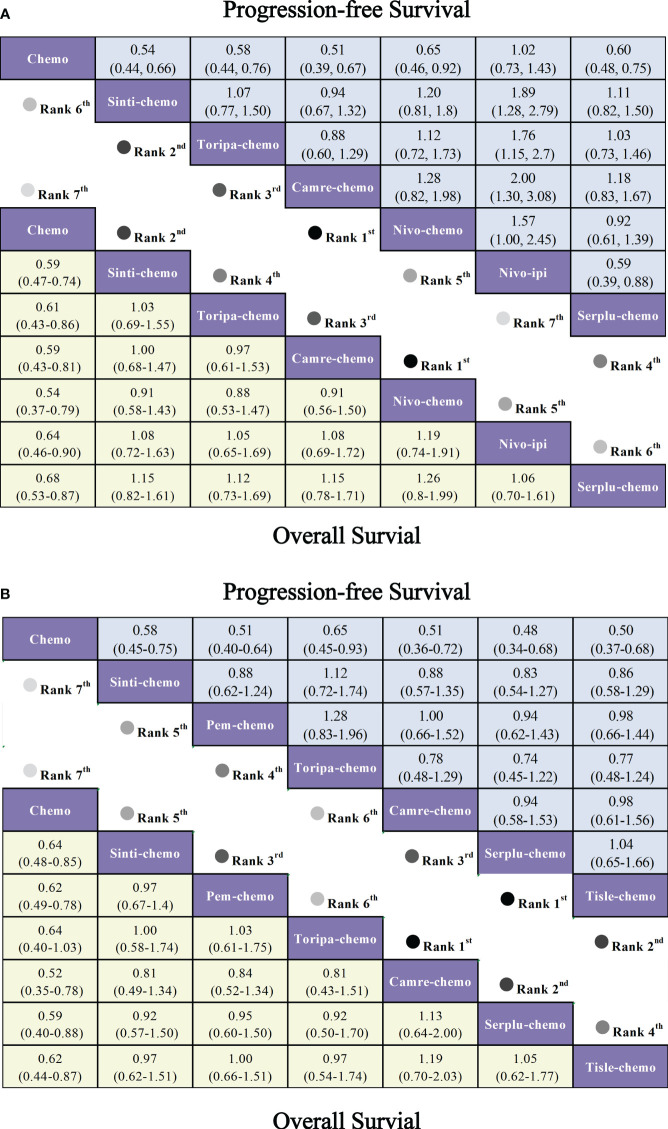
Forest plot comparing the efficacy and safety of various immunotherapy regimens in late-stage ESCC patients with PD-L1 expression ≥1% and PD-L1 expression ≥10% based on Bayesian network meta-analysis. **(A)** Risk ratios and 95% CIs for OS (depicted by the yellow lower triangle area) and PFS (depicted by the blue upper triangle area) in late-stage ESCC patients with PD-L1 expression ≥1%. HR < 1.00 indicates superior survival benefit. **(B)** Risk ratios and 95% CIs for OS (depicted by the yellow lower triangle area) and PFS (depicted by the blue upper triangle area) in late-stage ESCC patients with PD-L1 expression ≥10%. HR < 1.00 indicates superior survival benefit.

**Figure 10:**
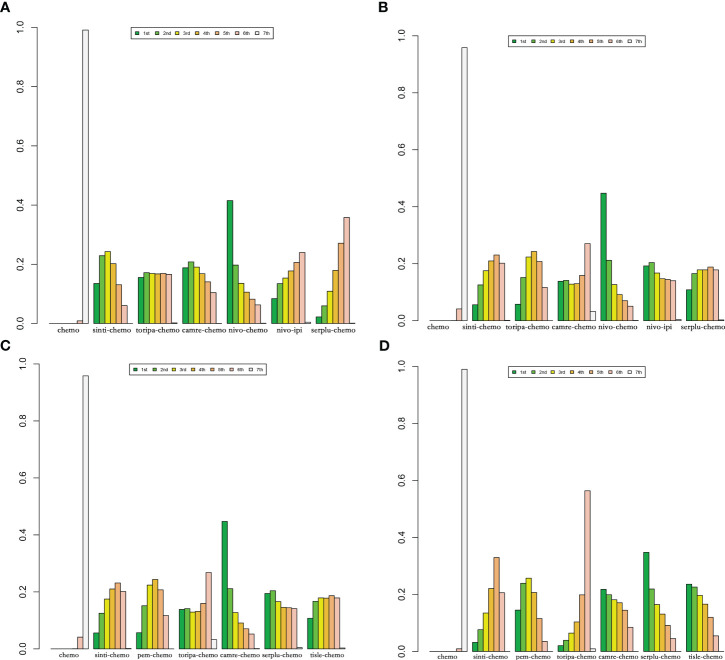
Bayesian ranking features of efficacy of various immunotherapy regimens in late-stage ESCC patients with PD-L1 expression ≥1% and PD-L1 expression ≥10%. **(A)** OS benefit ranking in advanced ESCC patients with PD-L1 expression ≥1%. **(B)** PFS benefit ranking in advanced ESCC patients with PD-L1 expression ≥1%. **(C)** OS benefit ranking in advanced ESCC patients with PD-L1 expression ≥10%. **(D)** PFS benefit ranking in advanced ESCC Patients with PD-L1 expression ≥10%.

In patients with PD-L1 expression ≥1%, six IO combinations were included in the subgroup analysis (**Figure 5C**). Regarding OS (**Figures 9A**, **10A**), all ICIs combination therapies showed OS benefits compared to chemotherapy in patients with PD-L1 ≥1%. Notably, nivo-chemo (HR=0.54, 95% CI: 0.37-0.79), sinti-chemo (HR=0.59, 95% CI: 0.47-0.74), and camre-chemo (HR=0.59, 95% CI: 0.43-0.81) significantly prolonged OS compared to chemotherapy. In terms of PFS (**Figures 9A**, **10B**), all treatment regimens except nivo-ipi showed PFS benefits compared to chemotherapy. Specifically, camre-chemo (HR=0.51, 95% CI: 0.39-0.67), sinti-chemo (HR=0.54, 95% CI: 0.44-0.66), and toripa-chemo (HR=0.58, 95% CI: 0.44-0.76) significantly extended PFS compared to chemotherapy.

In patients with PD-L1 expression ≥10%, six ICIs combinations were included in the subgroup analysis (**Figure 5D**). Overall, patients derived greater OS benefits from ICIs combination chemotherapy compared to standard chemotherapy. Among these combinations, camre-chemo (HR=0.52, 95% CI: 0.35-0.78) and serplu-chemo (HR=0.59, 95% CI: 0.40-0.88) were most likely to offer optimal OS benefits. Additionally, pem-chemo (HR=0.62, 95% CI: 0.49-0.78), tisle-chemo (HR=0.62, 95% CI: 0.44-0.87), sinti-chemo (HR=0.64, 95% CI: 0.48-0.85), and toripa-chemo (HR=0.64, 95% CI: 0.40-1.03) significantly prolonged OS compared to chemotherapy (**Figures 9B**, **10C**). In terms of PFS, all ICIs combination chemotherapies provided PFS benefits compared to standard chemotherapy. Serplu-chemo (HR=0.48, 95% CI: 0.34-0.68) and tisle-chemo (HR=0.50, 95% CI: 0.37-0.68) were most likely to offer optimal PFS benefits. Furthermore, camre-chemo (HR=0.51, 95% CI: 0.36-0.72), pem-chemo (HR=0.51, 95% CI: 0.40-0.64), sinti-chemo (HR=0.58, 95% CI: 0.45-0.75), and toripa-chemo (HR=0.65, 95% CI: 0.45-0.93) significantly prolonged PFS compared to chemotherapy (**Figures 9B**, **10D**).

### 3.6 Heterogeneity and inconsistency

The results of the pairwise meta-analysis based on the frequentist approach are consistent with the corresponding summary results in the Bayesian framework (**Supplementary Table 3**). Heterogeneity was assessed using the Q-test and *I^2^* statistic, showing low heterogeneity with *I^2^ =* 0% or *I^2^ ≤* 50% (**Figures 2**–**4**). As there was no high heterogeneity detected in this study, sensitivity analysis was not conducted. Funnel plots were used to analyze publication bias with OS as the outcome measure, revealing symmetrical distribution of study scatter points without any scattered distribution of study points, suggesting minimal potential for publication bias in this study (**Supplementary Figure 2**).

## 4 Discussion

As far as we are aware, the present study is the most comprehensive systematic review and network meta-analysis to date. It specifically examines and compares the effectiveness and safety of leading immune combination therapies. The findings of this study furnish robust evidence to inform clinical decision-making, delineating several pivotal insights:

(1) In general, treatment plans that include immune combination therapies have been linked to notable enhancements in OS and PFS, in comparison to traditional chemotherapy approaches.
(2) Among patients with advanced ESCC not selected based on PD-L1 expression, the combination of toripalimab with chemotherapy (toripa-chemo) and sintilimab with chemotherapy (sint-chemo) emerged as the most efficacious in terms of OS. Simultaneously, sintilimab combined with chemotherapy (sinti-chemo) and camrelizumab combined with chemotherapy (camre-chemo) showed superior PFS outcomes.
(3) In the subset of patients exhibiting PD-L1 expression levels of ≥1%, sintilimab combined with chemotherapy (sinti-chemo) and camrelizumab combined with chemotherapy (camre-chemo) yielded the most favorable OS and PFS outcomes, with nivolumab combined with chemotherapy (nivo-chemo) attaining the highest OS. For those with a PD-L1 expression of ≥10%, camrelizumab combined with chemotherapy (camre-chemo) and serplulimab combined with chemotherapy (serplu-chemo) were correlated with the optimal OS and PFS, respectively.
(4) Despite a higher incidence of toxicity associated with immune combination chemotherapy relative to standard treatment modalities, camrelizumab combined with chemotherapy (camre-chemo) demonstrated a better safety profile (HR=0.83, 95% CI: 0.59-1.16).
(5) Notably, camre-chemo achieves an optimal balance between therapeutic efficacy and tolerability, ranking favorably for OS, leading for PFS, and exhibiting the lowest incidence of Grade≥3 adverse events among all evaluated regimens in patients without PD-L1 expression preselection.

The observed superiority of immune combination chemotherapy over traditional regimens is likely attributable to their synergistic mechanisms of action. Antibodies targeting PD-1 interrupt the binding between PD-1 and its ligands, PD-L1 and PD-L2, effectively hindering immune evasion. Conversely, cytotoxic agents differ from ICIs in their capacity to modulate immune responses; for instance, fluorouracil and paclitaxel variably influence dendritic cell maturation and the eradication of myeloid-derived suppressor cells within the tumor microenvironment, cumulatively bolstering T cell-mediated anti-neoplastic effects. This dual action potentiates a more robust antitumor response (26–28).

Before the advent of immunotherapy regimens, fluorouracil and platinum-based chemotherapy served as the standard first-line treatment for advanced ESCC. However, the improvement in OS for ESCC patients was minimal (29). In 2021, the KEYNOTE-590 trial, published in The Lancet, made a significant breakthrough by comparing ICIs combined with chemotherapy directly to chemotherapy alone. Patients with ESCC who received pembrolizumab plus chemotherapy and had a PD-L1 expression of 10 or more achieved a median survival of 13.9 months, compared to 8.8 months with traditional chemotherapy alone. The results of this landmark study demonstrated that pembrolizumab plus chemotherapy significantly prolonged OS and should be recommended as a first-line treatment for patients with PD-L1 expression of 10 or more. Over the past three years, various large-scale RCTs have produced results indicating that multiple ICIs combined with chemotherapy regimens can be chosen for the treatment of advanced ESCC patients without PD-L1 selection, with PD-L1 expression ≥1 or PD-L1 expression ≥10.

It is important to acknowledge, however, that the overall safety profile of ICI plus chemotherapy regimens does not distinctly surpass that of chemotherapy alone in terms of treatment-related AEs graded 3 or above. The principal augmentation in adverse events attributable to the addition of ICIs is related to immune-mediated reactions. This observation underscores the manageable nature of the safety profile associated with ICI plus chemotherapy regimens, despite the increased incidence of immune-mediated AEs.

The therapeutic landscape for various malignancies has been markedly transformed with the advent of combination immune checkpoint inhibitor (ICI) therapies, notably the synergistic pairing of anti-PD-1 agents (nivolumab) with anti-CTLA-4 agents (ipilimumab). This combination has received formal endorsement from the U.S. Food and Drug Administration (FDA) for a spectrum of cancers, including melanoma, renal cell carcinoma, colorectal cancer, hepatocellular carcinoma, non-small cell lung cancer, and notably, ESCC (16, 30–34). The combination of Nivolumab and ipilimumab is recommended as an initial treatment for esophageal squamous cell cancer, corroborated by the CheckMate-648 study’s results. The study showed that the combination of Nivolumab and ipilimumab enhances the overall survival of ESCC patients, surpassing the effects of sole chemotherapy. This research found that nivolumab and ipilimumab placed 7th in overall survival rates among treatment methods in the PD-L1 general population and 5th in those with PD-L1 expression of 1% or more, surpassing the efficacy of chemotherapy. Although Nivolumab plus ipilimumab did not rank significantly superior in efficacy, it ranked second in terms of grade 3 or higher AEs safety indicators among all treatment groups and was superior to chemotherapy. Nivolumab plus ipilimumab belongs to the category of ICIs combination therapy, exhibiting a synergistic effect. The two ICI drugs target PD-1 receptor and CTLA-4 receptor respectively; ipilimumab activates and proliferates T cells, while nivolumab enables T cells to recognize tumor cells. The synergistic action of these two mechanisms aids in tumor cell elimination. According to our research results, the incidence of grade 3 or higher treatment-related AEs with nivolumab plus ipilimumab was lower than that with chemotherapy. As the inclusion of chemotherapy may significantly increase toxicity, future head-to-head comparative studies between nivo-ipi and nivo-ipi-chemo are needed to better assess the safety of nivo-ipi.

PD-L1 expression status may serve as a biomarker for predicting the clinical efficacy of ICIs in the treatment of various malignancies (35). Therefore, to better evaluate the clinical efficacy of ICIs for ESCC, we included not only PD-L1 unselected ESCC patients but also those with PD-L1 expression ≥10% and PD-L1 expression ≥1%. Studies have shown that both PD-L1 expression ≥1% and PD-L1 expression ≥10% ESCC patients experience significant OS and PFS benefits with ICIs compared to traditional chemotherapy. Thus, PD-L1 expression status can be used to predict the clinical efficacy of ICIs in treating ESCC.

This study synthesizes data from large-scale RCTs to assess the efficacy and safety of various first-line immunotherapy regimens for advanced ESCC, providing a reference for clinical medication selection. When considering clinical efficacy and safety, camrelizumab combined with chemotherapy (camre-chemo) emerges as an outstanding first-line therapeutic option for advanced ESCC patients without selected PD-L1 expression. This finding is consistent with Gao’s meta-analysis (36). However, our study incorporates a more comprehensive selection of RCTs, and we also specifically address treatment options for patients with PD-L1 positive ESCC. Our findings indicate that integrating ICIs with chemotherapy increases toxicity compared to traditional chemotherapy alone. Furthermore, our results demonstrate that for patients with varying degrees of PD-L1 positive expression in advanced ESCC, selecting the appropriate first-line immunotherapy regimen can lead to improved survival outcomes. This could assist guidelines in addressing the optimal treatment strategy for advanced ESCC patients based on different PD-L1 expression levels.

Future studies necessitate more head-to-head comparisons, such as between nivolumab plus ipilimumab (nivo-ipi) and nivo-ipi combined with chemotherapy, to better evaluate the safety and effectiveness of treatment regimens. Although the studies included in our analysis are large multicenter RCTs featuring diverse ethnic groups, we observed that over 80% of the participants were of Asian descent, with a minimal representation of Black patients. Given the high incidence of ESCC in African populations, increasing the inclusion of Black patients in future RCTs would enhance the representativeness and comprehensiveness of the research findings.

This study elucidates several pivotal insights into the first-line immunotherapy regimens for advanced ESCC. However, it is imperative to consider a few limitations that may impact the interpretation and generalizability of our findings. First, there was variability in the standard chemotherapy regimens employed across the analyzed studies. For instance, the JUPITER-06 and ESCORT-1st trials favored a paclitaxel and cisplatin combination, whereas Checkmate 648 and ASTRUM-007 utilized a regimen of fluorouracil with cisplatin. Despite both being recognized standards for first-line chemotherapy, such discrepancies could potentially introduce a bias in the comparative analysis. Secondly, due to the limited number of RCTs focusing on ESCC, many interventions had only one randomized controlled trial, which may limit the generalizability of our conclusions. Thirdly, the included studies employed two methodologies to evaluate PD-L1 expression: Tumor Proportion Score (TPS), defined as the percentage of PD-L1 positive tumor cells out of the total number of viable tumor cells, and Combined Positive Score (CPS), which accounts for the ratio of PD-L1 expressing tumor cells, lymphocytes, and macrophages to the total number of viable tumor cells (37). Due to CPS’s more accurate depiction of the tumor microenvironment and PD-L1 expression status, our preference for assessing PD-L1 expression primarily relied on CPS. However, for studies like Checkmate648 and Escort1, which only utilized the TPS method, PD-L1 expression was assessed accordingly. The variance in PD-L1 expression assessment between these two methods may introduce certain biases. Lastly, as mentioned earlier, the incidence of ESCC is notably higher in Africa, yet the patient demographic included in our analysis comprised less than 5% Black individuals. This discrepancy raises questions regarding the applicability of our study’s conclusions to the Black population. Given that over 90% of advanced ESCC patients are of Asian descent, despite our exhaustive search, the studies included in our analysis provide minimal data on Caucasian populations, preventing further analysis based on racial differences. Consequently, we must acknowledge that the conclusions drawn from this study are more applicable to Asian populations.

Despite these limitations, our research offers a comprehensive summary of randomized controlled trials on first-line immunotherapy for advanced esophageal squamous carcinoma, shedding light on significant insights and guiding future clinical practices.

## Data availability statement

The original contributions presented in the study are included in the article/**Supplementary Material**. Further inquiries can be directed to the corresponding author.

## Author contributions

WC: Conceptualization, Data curation, Writing – original draft, Writing – review & editing. KC: Data curation, Software, Writing – original draft, Writing – review & editing. LZ: Data curation, Software, Writing – review & editing. XZ: Supervision, Validation, Writing – review & editing. BC: Formal analysis, Writing – review & editing. WL: Data curation, Visualization, Writing – review & editing. RS: Validation, Writing – review & editing. LS: Data curation, Writing – review & editing. ZJ: Supervision, Validation, Writing – review & editing. JW: Validation, Writing – review & editing. WX: Methodology, Supervision, Writing – original draft, Writing – review & editing.
